# Plasma Asprosin Levels Are Associated with Glucose Metabolism, Lipid, and Sex Hormone Profiles in Females with Metabolic-Related Diseases

**DOI:** 10.1155/2018/7375294

**Published:** 2018-11-06

**Authors:** Xing Li, Mingyu Liao, Rufei Shen, Linlin Zhang, Hua Hu, Jun Wu, Xiuli Wang, Hua Qu, Shaodong Guo, Min Long, Hongting Zheng

**Affiliations:** ^1^Department of Endocrinology, Xinqiao Hospital, Third Military Medical University (Army Medical University), Chongqing 400037, China; ^2^Department of Obstetrics and Gynecology, Xinqiao Hospital, Third Military Medical University (Army Medical University), Chongqing 400037, China; ^3^Department of Health Management, Xinqiao Hospital, Third Military Medical University (Army Medical University), Chongqing 400037, China; ^4^Department of Nutrition and Food Science College of Agriculture and Life Sciences, Texas A&M University, Texas 77843, USA

## Abstract

Asprosin is a white adipose tissue-derived hormone that increases abnormally in mammals with insulin resistance. However, the role of asprosin in polycystic ovary syndrome (PCOS), a disease partly characterized by insulin resistance, and its potential connection with type 2 diabetes mellitus (T2DM) and PCOS has not been thoroughly elucidated to date. To investigate the association of asprosin with metabolic profiles, sex-related hormones, or inflammation in females with T2DM or PCOS, plasma asprosin and metabolic indicators were measured in 66 healthy females, 53 female patients with T2DM, and 41 patients with PCOS. Spearman's correlation analysis and binary logistic regression analysis models were used. Plasma asprosin was significantly higher in T2DM females than in healthy subjects (*P* < 0.001) and was positively correlated with fasting blood glucose (FBG), hemoglobin A1c (HbA1c), and HOMA-IR (*P* < 0.05). Asprosin in PCOS subjects was also higher than in healthy subjects (*P* < 0.001) but lower than in T2DM subjects (*P* < 0.05), and it was positively correlated with FBG, HbA1c, HOMA-IR, LDL-c, APOB, APOE, and testosterone (*P* < 0.05). The BMI-categorized subgroups of PCOS subjects also showed correlations of asprosin with metabolic profiles and sex-related hormones. Binary logistic regression analysis revealed that plasma asprosin level acted as an independent risk factor for T2DM or PCOS. These findings suggest the correlation of plasma asprosin level with glucose metabolism, lipid metabolism, sex-related hormones, and inflammation in females, supporting asprosin as a potential predictive factor for females with metabolic-related diseases. This trial is registered with ChiCTR-ROC-17010719.

## 1. Introduction

Type 2 diabetes (T2DM) is a multifaceted disease involving the pancreatic *β*-cells, adipose tissue, liver, and other organs [1]. Hyperglycemia, insulin resistance (IR), and obesity are characteristic features of T2DM and play crucial parts in the pathogenesis of the disease [2]. Adipose tissue, which produces adipocytokines that modulate insulin sensitivity and inflammation, is the primary site where IR manifests [3, 4]. These features of T2DM also exist in some patients with polycystic ovary syndrome (PCOS), a metabolic and reproductive disorder primarily featured by hyperandrogenism and ovarian dysfunction [5]. PCOS patients most commonly have postprandial dysglycemia, reflecting peripheral IR and conferring increased risk of T2DM, mainly in obese women [6, 7]. Thus, how obesity-related factors originating from adipose tissue affect females with metabolic-related diseases, such as PCOS or T2DM, needs to be clarified.

Asprosin has recently been identified as a fasting-induced glucogenic hormone secreted by white adipose tissue (WAT) that promotes hepatic glucose production [8]. It is also an orexigenic hormone that activates AgRP neurons to increase food consumption and body weight [9]. Asprosin is pathologically elevated in human and mouse with IR or obesity [8]. Conversely, a genetic deficiency of asprosin leads to neonatal progeroid syndrome (NPS), characterized by low appetite and extreme leanness [9]. A single injection of asprosin causes a swift rise in blood glucose and insulin in mice, similar to humans, while a reduction in asprosin improves insulin sensitivity and reduces appetite and body weight in mice [8]. Recent studies found that asprosin is increased in T2DM patients, which is associated with fasting glucose and triglycerides (TG) [10].

To gain insight into the function of plasma asprosin in females, a cross-sectional study was conducted to evaluate whether asprosin correlates with glucose homeostasis, IR, obesity, lipid metabolism, inflammation markers, and even sex hormones in females with T2DM or PCOS. In this study, we analyzed anthropometric, metabolic, and hormonal predictors, as well as circulating asprosin level, in 66 healthy females, 53 females with newly diagnosed T2DM, and 41 females with PCOS. We also compared plasma asprosin in the body mass index- (BMI-) divided normal-weight and overweight/obese subgroups of T2DM or PCOS patients to detect correlations between asprosin and metabolic diseases in females.

## 2. Materials and Methods

### 2.1. Study of Healthy, T2DM, and PCOS Subjects

The clinical trial ran from February 2017 to December 2018. We recruited 160 female subjects, including 53 patients newly diagnosed with T2DM, 41 subjects with PCOS, and 66 healthy females. The patients in these three groups were categorized into subgroups as normal-weight (BMI < 24 kg/m^2^) and overweight/obese (BMI ≥ 24 kg/m^2^), according to BMI [11]. Eleven subjects in the T2DM group and 3 in the PCOS group had a medication history of antidiabetic, antihypertension, or antihyperlipidemia drugs (Supplementary Table 1). As supplementary data, we excluded these subjects who used these drugs and analyzed the data again with the rest of the subjects (Supplementary Figure 1, Supplementary Tables 2–7).

The diagnosis of T2DM referred to diagnostic criteria from the World Health Organization [12]. T2DM subjects were newly diagnosed without hypoglycemic or dietary treatment. In accordance with the Rotterdam criteria [13], PCOS was diagnosed with the presence of two of the following features, hyperandrogenism and oligo- and/or anovulation, and polycystic ovaries examined by ultrasound, with the exclusion of androgen-secreting tumors, hyperprolactinemia, 21-hydroxylase deficiency (21-OHD), Cushing's syndrome, congenital adrenal hyperplasia, thyroid disease, and other diseases. Abnormal intrauterine cavity, a history of recurrent spontaneous abortion, and unilateral oophorectomy were also excluded. The other exclusion criteria were type 1 diabetes, malignant diseases, hypertension, symptomatic heart failure, hepatic failure, and renal failure.

The control group was recruited from those who underwent a routine medical checkup, pelvic examination, and ultrasonography and had normal FBG lower than 6.1 mmol/liter, 2-hour oral glucose tolerance test (2 h-OGTT) glucose lower than 7.8 mmol/liter, no family history of T2DM, normal menstrual cycle, no clinical or biochemical hyperandrogenism, and no medications (antidiabetic drugs, antihypertension drugs, antiobesity drugs, antiandrogens, oral contraceptives, insulin sensitizers, glucocorticoids, and ovulation induction agents) that could affect metabolic or sex-related hormones for at least half a year before the study. This study was authorized by the Human Research Ethics Committee of Xinqiao Hospital. Informed consent was procured from all subjects.

### 2.2. Measurement of Plasma Asprosin

Plasma asprosin was determined by the ELISA kit from Abbexa Ltd. (Catalog No: abx257694; Abbexa, Cambridge, UK). The kit had a sensitivity of 0.938 ng/mL, with a range between 1.563 ng/mL and 100 ng/mL. The intra-assay and interassay variations were, respectively, 8% and 10%.

### 2.3. Anthropometric and Biochemical Measurements

Blood samples were acquired after an overnight fast. Samples from PCOS patients were obtained at the follicular phase of the menstrual period. Blood glucose and glycosylated hemoglobin A1c (HbA1c) were measured by the glucose-oxidase method and anion-exchange high-performance liquid chromatography (HPLC), respectively. Serum insulin was measured by radioimmunoassay. The homeostasis model assessment of IR (HOMA-IR) and insulin secretion (HOMA-*β*) were calculated using the following equations [14]: HOMA − IR = fasting insulin (FINS) (microunits/milliliter) × FBG (millimoles/liter)/22.5 and HOMA − *β* = [20 × FINS (microunits/milliliter)]/[FBG (millimoles/liter) − 3.5]. BMI was calculated by weight/height2 (kg/m^2^). C-reactive protein (CRP), total cholesterol (TC), TG, high-density lipoprotein cholesterol (HDL-C), and low-density lipoprotein cholesterol (LDL-C) were determined using an enzymatic autoanalyzer (Hitachi 747; Hitachi, Tokyo, Japan). Tumor necrosis factor- (TNF-) *α*, interleukin- (IL-) 6, and IL-8 were measured by IMMULITE 1000 Immunoassay System (Siemens Healthcare Diagnostics Inc.). The liver and renal function profiles were measured by an autoanalyzer (ARCHITECT c16000 System, Abbott Laboratories, Lake Bluff, Illinois, USA). Sex hormones, including luteinizing hormone (LH), follicle-stimulating hormone (FSH), testosterone, estradiol, progesterone, and prolactin, were determined by the IMMULITE 2000 Immunoassay System (Siemens Healthcare Diagnostics Inc). Serum sex hormone-binding globulin (SHBG) was determined by immunoradiometric assays. Dehydroepiandrosterone sulfate (DHEA-S), 17-*α*-hydroxyprogesterone (17*α*-OHP), and anti-Mullerian hormone (AMH) were measured by RIA (Diagnostic Systems Laboratories).

### 2.4. Oral Glucose Tolerance Test (OGTT) and Insulin Release Test

After an overnight fast, OGTTs and insulin release tests were conducted in 29 T2DM females and 41 PCOS subjects. In accordance with the World Health Organization guidelines, patients ingested 75 g glucose in 5 min, and we measured blood insulin before the start (0 min) and at 30, 60, 120, and 180 min, and then the area under the insulin concentration-time curve (IAUC) was calculated.

### 2.5. Euglycemic-Hyperinsulinemic Clamp Test (EHCT)

EHCT was performed in 17 young women with PCOS between 14 and 29 years of age. Briefly, after an overnight fast, insulin and glucose were infused by a catheter in an antecubital vein while venous blood was withdrawn from the other hand. Euglycemia and hyperinsulinemia were maintained by glucose infusion with a variable rate and continuously insulin (1 mU/kg/min) infusion, respectively. The blood insulin concentrations were recorded before the start (0 min) and at 90 min and at 180 min.

### 2.6. Statistical Analysis

Analyses were conducted by IBM SPSS Statistics 24.0. Comparisons among the three groups were performed by analysis of variance (ANOVA); the least significant difference and Student-Newman-Keuls tests were used as post hoc tests. Normally distributed continuous variables are described as mean ± standard deviation, and between-group differences were compared by an unpaired *T* test. For nonnormally distributed continuous variables, medians (25–75 percentiles) are presented, and comparisons were done by the Mann-Whitney *U* test. Interrelationships between variables were assessed using Spearman's correlation analysis and a partial correlation analysis adjusted for age. The association of asprosin with T2DM or PCOS was examined by binary logistic regression analysis. The adjusted odds ratio (OR) and 95% confidence interval (CI) are presented. *P* < 0.05 was defined as statistically significant.

## 3. Results

### 3.1. Abnormal Change in Metabolic Profiles or Sex-Related Hormones in the T2DM or PCOS Groups

As shown in Table 1, the average age of participants in the newly diagnosed T2DM group was older than that of the PCOS group due to disease incidence. BMI and waist-to-hip ratio (WHR) were significantly higher in T2DM and PCOS females than in healthy subjects (*P* < 0.01), which suggested the role of metabolic abnormality and obesity in these two diseases. Systolic blood pressure (SBP) was higher in T2DM subjects than in healthy or PCOS subjects (*P* < 0.01), probably owing to the older age of the T2DM patients. Glucose and insulin-metabolic parameters, including FBG, FINS, HbA1c, and HOMA-IR, were all significantly higher (58.95%, 32.74%, 59.17%, and 110.12%, respectively) in T2DM subjects than in healthy subjects; the average levels of FBG, HbA1c, and HOMA-IR in the T2DM group were the highest among the three groups (*P* < 0.05 or *P* < 0.01). PCOS subjects showed the highest average FINS and HOMA-*β* values (*P* < 0.01), revealing that female patients with PCOS had obvious IR and compensatory hyperinsulinemia. In the lipid profiles, significantly higher TG, TC, and LDL-C, along with lower HDL-c, were found in T2DM and PCOS subjects than in healthy subjects (*P* < 0.05 or *P* < 0.01). Liver enzymes, including glutamic-pyruvic transaminase (ALT), glutamic-oxaloacetic transaminase (AST), and *γ*-glutamyltransferase (*γ*-GGT), as well as renal function indexes, including creatinine (Cre) and blood urea nitrogen (BUN), seldom showed significant differences between groups, except for the obviously elevated uric acid (UA) in PCOS patients. Among inflammatory markers, WBC was significantly higher in the T2DM and PCOS groups (*P* < 0.01), while CRP was higher in the T2DM group compared to PCOS (*P* < 0.01), indicating the chronic inflammation state in T2DM and PCOS patients. With regard to sex hormones, PCOS patients showed excess androgen levels and a lack of periodic female hormone secretion, such as significantly higher testosterone and LH/FSH (42.7% and 23.93% higher, respectively), as well as lower estradiol and progesterone, compared to those in healthy subjects (38.94% and 62.93% lower, respectively) (*P* < 0.001).

As the overweight/obese state of the body is closely related to WAT-derived asprosin [8], as well as the pathogenesis of T2DM and PCOS [7, 15], we stratified these groups into subgroups by BMI (≥24 kg/m^2^ was considered overweight/obese) [11]. In the newly diagnosed T2DM females, only WHR and OGTT-180 min insulin differed significantly between the subgroups (*P* < 0.05 or *P* < 0.001; Table 2). However, in PCOS subjects, other than BMI, variables related to glucose homeostasis and IR (e.g., FINS, HbA1c, HOMA-IR, OGTT-60 min insulin, OGTT-120 min insulin, and IAUC), as well as TC and liver enzymes (ALT, *γ*-GGT), were all significantly higher in the overweight/obese subgroup than the normal-weight subgroup (*P* < 0.05 or *P* < 0.01). We also found that sex hormones in PCOS did not significantly differ between the BMI-categorized subgroups (data not shown). When excluding the subjects who has used drugs (antidiabetic, antihyperlipidemia, and antihypertensive drugs), the results are consistent with the previous notion that subjects in T2DM and PCOS groups, especially in overweight subgroups, show abnormal glucose metabolism, as well as sex hormone dysfunction in PCOS females (Supplementary Tables 2 and 3).

### 3.2. Plasma Asprosin Levels in the Healthy, T2DM, and PCOS Groups and the BMI-Categorized Subgroups of T2DM and PCOS

**Figure 1:**
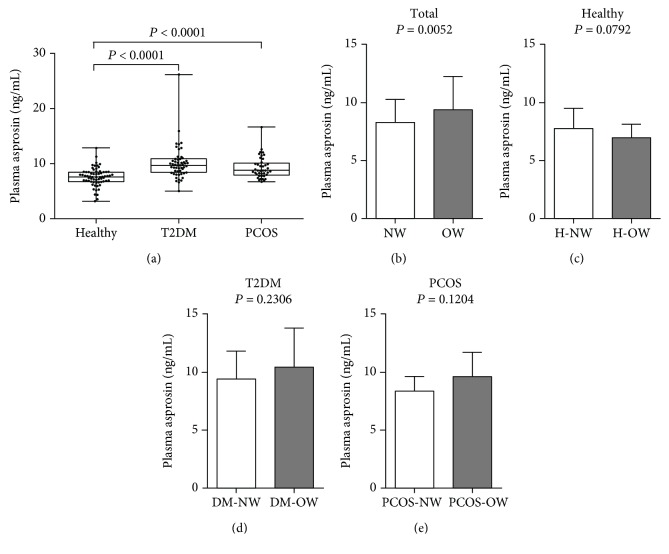
Plasma asprosin concentrations in groups and the overweight/obese subgroups. (a) *n* = 66 healthy, 53 T2DM, and 41 PCOS female subjects. *P* < 0.0001 compared with the healthy group. No significant difference was found between the T2DM and PCOS groups. (b) *n* = 82 NW and 78 OW subjects from all groups. (c) *n* = 49 NW and 17 OW from the healthy group. (d) *n* = 21 NW and 32 OW from the T2DM group. (e) *n* = 12 NW and 29 OW from the PCOS group. [Normal weight (NW) was defined as BMI < 24 kg/m^2^, and overweight/obesity (OW) was defined as BMI ≥ 24 kg/m^2^]. Data are presented as means ± SD, and unpaired *T* tests were performed.

Plasma asprosin was significantly higher in T2DM and PCOS patients than in the healthy subjects, and it was highest in the T2DM group (both *P* < 0.001; Figure 1(a)). However, there was no significant difference between the T2DM and PCOS groups. Moreover, asprosin in overweight/obese females with T2DM or PCOS seemed to be slightly higher than in normal-weight patients, but no difference was observed between the two BMI-categorized subgroups of healthy subjects (Figures 1(c)–1(e)). In addition, the deviation of the selected subject populations may partly explain the difference in the overall results: plasma asprosin in the overweight subgroup of the total subject group was higher than in the normal-weight subgroup (*P* < 0.01; Figure 1(b)). Excluding subjects who have used drugs did not influence the significant difference between groups (Supplementary Figure 1).

### 3.3. Association of Plasma Asprosin Level with Metabolic Profile in All Subjects, the T2DM Group, and the BMI-Divided T2DM Subgroup

To investigate the associations of circulating asprosin concentration with metabolic parameters in all subjects of this study, Spearman's correlation analysis was conducted. Our data showed that plasma asprosin concentration was positively correlated with glucose metabolic factors (e.g., FBG, FINS, HbA1c, and HOMA-IR), lipid factors (e.g., TG, TC, and LDL-c), basal indicators (e.g., BMI, WHR, SBP, and DBP), and WBC after adjusting for age (*P* < 0.05 or *P* < 0.01; Table 3).

Considering the potential association between asprosin and predictors in T2DM females, the age factor was adjusted, and all T2DM subjects were divided into two subgroups according to BMI. We found that plasma asprosin constantly had positive correlations with glucose metabolism factors, including FBG and HbA1c (*P* < 0.05 or *P* < 0.001; Table 4) in both overall group and two subgroups. Moreover, asprosin level was positively correlated with HOMA-IR in T2DM and its normal-weight subgroup (*P* < 0.05 or *P* < 0.01) and negatively correlated with OGTT-120 min insulin in the normal-weight subgroup and with HDL-C in the overweight subgroup (*P* < 0.05). We conclude that plasma asprosin level was age-independently correlated with glucose metabolism and homeostasis, especially in the T2DM group as well as its subgroups categorized by body mass. In addition, the binary logistic regression analysis revealed that plasma asprosin level was significantly correlated with T2DM in a model after controlling for age, BMI, BPs, the inflammatory marker WBC, lipid profile, and liver function factors, including ALT, AST, and *γ*-GGT [OR: 2.790, 95% CI: 1.132–6.874; *P* = 0.026], suggesting that asprosin could be an independent risk factor for T2DM (Table 5).

### 3.4. Associations of Plasma Asprosin Level with Metabolic Profile and Sex-Related Hormones in PCOS Females, including the BMI-Divided Subgroups

Next, we evaluated the associations between plasma asprosin level and metabolic factors and sex-related hormones in PCOS patients. After adjusting for age, plasma asprosin level was positively correlated with factors related to glucose metabolism (i.e., FINS, HOMA-IR, and HbA1c), lipid factors (i.e., LDL-c, APOE, and APOB), and testosterone (*P* < 0.05 or *P* < 0.01; Table 6) in all PCOS subjects and the overweight/obese subgroup. Asprosin level was negatively correlated with prolactin (*P* < 0.05). These findings coincide with the notion that obesity-induced hyperinsulinemia stimulates androgen production [16–18] and that the interactions between prolactin and adipokines also affect global metabolism [19, 20]. In addition, in normal-weight PCOS subjects, asprosin still had a positive correlation with estradiol secreted by ovarian granulocytes but was negatively correlated with SHBG, a transporter for most of the estradiol in blood (*P* < 0.05 or *P* < 0.01), suggesting that asprosin level might be an indirect trigger or just an indicator of metabolic and sex-related hormone dysfunction. Moreover, we failed to find any correlation between asprosin and inflammatory markers (i.e., WBC, CRP, TNF-*α*, IL-6, and IL-8). In addition, binary logistic regression analysis revealed that plasma asprosin level could also be an independent risk factor for PCOS, as it was significantly correlated with PCOS in a model adjusted for age, BMI, BPs, WBC, and lipid profiles (OR: 2.483, 95% CI: 1.102–5.596; *P* = 0.028) (Table 5). After excluding subjects who have used drugs, the statistical results are consistent with our previous notion that asprosin is associated with glucose metabolism, lipid metabolism, inflammation, and sex hormone homeostasis (Supplementary Tables 4–7).

## 4. Discussion

As a novel protein hormone enriched in WAT, blood asprosin abnormally increases in the blood of mammals with IR [8, 9]. IR is implicated in inflammation, contributing to the pathogenesis of some metabolic-related diseases, such as T2DM and PCOS [21, 22]. Increased asprosin has been independently correlated with FBG and TG in T2DM. However, knowledge regarding the function of asprosin in PCOS patients or in the potential connection between T2DM and PCOS remains lacking. In this research, we demonstrated that plasma asprosin was significantly higher in both PCOS and T2DM female patients than in healthy subjects. Moreover, overweight participants from all the groups, regardless of T2DM or PCOS status, showed significantly higher asprosin than did lean individuals. Our results from humans are consistent with the observation in animals by Romere et al. [8] and Duerrschmid et al. [9]. Other studies suggested that asprosin promotes hepatic glucose production via cAMP [8] and that circulating asprosin can cross the blood-brain barrier, then activate orexigenic AgRP-positive neurons via a cAMP-dependent pathway, consequently inhibiting anorexigenic proopiomelanocortin- (POMC-) positive neurons, which stimulates appetite and results in adipose accumulation [9]. Recently, several cross-sectional studies have shown an association between plasma asprosin level and the pre-DM and T2DM states [10, 23].

Our data in females showed that plasma asprosin concentration was closely associated with body mass, glucose metabolism, insulin secretion, and inflammation in all participants, including healthy and diseased groups, as asprosin level was significantly correlated with profiles of adiposity, glucose and insulin metabolism, and WBC level, even after adjusting for age. Previous studies also revealed that plasma asprosin upregulation by the overexpression of its encoding gene, fibrillin-1, in WAT seemed unique to the pathogenesis of IR [8]. Our subgroup analysis discovered that the average plasma asprosin level was obviously higher in overweight/obese subgroups. Moreover, in the age-adjusted T2DM groups, and even in the BMI-categorized T2DM subgroups, asprosin was still correlated with several predictors related to glucose, insulin, and lipid homeostasis. These results suggest that plasma asprosin might be a universal hormone marker as well as a risk factor to reflect glucose homeostasis, IR, or the obese state.

Since obesity and IR also prevail in PCOS patients, whether asprosin influences or reflects the occurrence and development of PCOS and how asprosin interacts with metabolic or sex hormones need to be clarified. We compared sex hormones between healthy and PCOS females and the metabolic profiles between subgroups of PCOS patients, finding that overweight/obesity, excess insulin and androgen secretion, and reduced estradiol and progesterone all had a higher prevalence in PCOS females. Although overweight PCOS subjects showed abnormal insulin, glucose, and lipid metabolism, no significant differences in sex hormones were detected between normal-weight and overweight/obese PCOS females, though these results might be limited by the smaller number of normal-weight PCOS subjects. Importantly, plasma asprosin level in the PCOS group was significantly correlated with glucose, insulin, and lipid metabolism profiles. However, in normal-weight PCOS females, these metabolic factors seldom showed correlations with plasma asprosin. Sex-related hormones such as estradiol and SHBG were significantly correlated with asprosin, which could confirm the notion that overweight/obesity confers IR and then worsens the reproductive and metabolic features of PCOS [15, 24, 25]. Moreover, in all the PCOS subjects and the overweight/obese subgroup of PCOS, asprosin was consistently correlated with testosterone and prolactin. Previous hypotheses could explain the positive correlation between asprosin and testosterone, as well as the negative correlation between asprosin and SHBG, since IR resulting in hyperinsulinemia could increase hyperandrogenism through insulin-induced ovarian steroidogenesis and inhibit hepatic SHBG production [26]. To our knowledge, even in PCOS, asprosin still can reflect the obese and IR states, which might also be mechanically related to the abnormal sex-related hormone metabolism in the pathogenesis with PCOS. Interestingly, several studies have traced the connections between obesity, IR, and sex hormone production. Other studies have reported that adipose tissue provides storage and a metabolic site for various fat-soluble steroids, including androgens, which contribute further to hyperandrogenism [27]. Further studies have revealed that insulin-driven androgen excess and dysfunctional lipid metabolism in the adipose tissue are causative drivers of metabolic risk in PCOS [28, 29]. Asprosin is derived from WAT and correlated with sex-related hormones in PCOS females, especially in overweight/obese subgroup of PCOS, indicating that asprosin might participate in the occurrence or development of PCOS, not only via obesity and IR but also by interacting with sex hormones.

Low-grade inflammation in obese subjects, which seems to be largely attributable to adipokines, is a condition associated with increased risk of metabolic and inflammatory diseases [30, 31]. We found that T2DM and PCOS female patients showed an inflammatory state, which is consistent with previous studies [21, 22]. Moreover, our data indicated that asprosin had correlations with inflammation. Specifically, asprosin was positively correlated with WBC in the total study population but was not correlated with other inflammation makers (e.g., CRP, TNF-*α*, IL-6, or IL-8), in the T2DM or PCOS group or their subgroups, while the results for inflammation markers (TNF-*α*, IL-6, or IL-8) in healthy control and T2DM subjects have been reported many times in literature [32–37]. Similarly, our previous research failed to find a correlation between plasma asprosin level and CRP [23]. However, in the hypomorphic fibrillin-1-mutant mouse model, blood asprosin decreased, while secretion of proinflammatory cytokines (e.g., IL-6, MCP-1, and GM-CSF) was enhanced in the ascending aorta [38, 39]. Thus, whether asprosin influences systematic inflammation in T2DM or PCOS patients still needs further study.

Though our investigation is limited by the cross-sectional design and a lack of detection of some inflammatory factors (TNF-*α*, IL-6, and IL-8), it demonstrated the potential correlations between this molecule and metabolic or sexual parameters in a limited number of participants. To investigate the mechanisms for this correlation, more detailed experiments on asprosin in the PCOS group are needed, such as experiments illuminating how asprosin communicates with sex-related hormones and inflammatory reactions. Additionally, further studies are needed to clarify whether plasma asprosin could be a vital predictor of PCOS.

## 5. Conclusions

This study analyzed the potential roles of plasma asprosin in healthy, T2DM, and PCOS females and demonstrated that plasma asprosin increased in patients with PCOS and was associated with various metabolic parameters and sex-related hormone profiles. Moreover, blood asprosin might play a vital role in glucose homeostasis, insulin homeostasis, obesity, sex-related hormone metabolism, or inflammation in females with metabolic-related diseases.

## Figures and Tables

**Table 1:** Clinical, metabolic, and sex hormone features of healthy, T2DM, and PCOS female subjects.

	Healthy	T2DM	PCOS	*F*/*t*/*Z*	*P*
Age (years)	37.02 ± 8.16	47.02 ± 4.92 ^b^	22.68 ± 5.66 ^b,d^	156.63	<0.001
BMI (kg/m^2^)	22.68 ± 4.00	24.98 ± 3.31 ^b^	26.68 ± 5.66^b,c^	16.38	<0.001
WHR	0.73 ± 0.04	0.89 ± 0.10 ^b^	0.90 ± 0.09^b^	90.51	<0.001
SBP (mmHg)	116.05 ± 9.57	125.06 ± 11.13 ^b^	118.10 ± 10.90^d^	11.45	<0.001
DBP (mmHg)	73.09 ± 7.82	77.00 ± 8.78 ^a^	76.13 ± 8.41	3.62	0.029
FBG (mmol/L)	4.75 ± 0.55	7.55 ± 2.97 ^b^	4.74 ± 2.22^d^	32.33	<0.001
FINS (mU/mL)	7.85 ± 5.94	10.42 ± 6.29 ^a^	14.62 ± 5.94 ^b,d^	15.77	<0.001
HbA1c (%)	5.51 ± 0.31	8.77 ± 2.44 ^b^	6.08 ± 1.69 ^d^	41.67	<0.001
HOMA-IR	1.68 ± 1.37	3.53 ± 2.53^b^	3.03 ± 1.55 ^b^	15.47	<0.001
HOMA-*β*	112.12 (78.49–157.88)	49.17 (28.954–105.07)	320.20 (162.31–457.08) ^b,d^	7.90	0.001
TC (mmol/L)	4.18 ± 0.76	4.74 ± 0.94^b^	4.47 ± 1.05	5.70	0.004
TG (mmol/L)	0.94 (0.73–1.25)	1.66 (1.26–2.31)^b^	1.22 (0.73–2.16)^a^	10.75	<0.001
LDL-C (mmol/L)	2.58 ± 0.64	3.18 ± 0.78^b^	2.86 ± 0.79^c^	9.91	<0.001
HDL-C (mmol/L)	1.43 ± 0.33	1.17 ± 0.27^b^	1.21 ± 0.39^b^	10.39	<0.001
AST (IU/L)	22.83 ± 16.16	21.99 ± 13.95	22.98 ± 11.60	0.06	0.939
ALT (IU/L)	18.64 ± 18.74	25.46 ± 18.05	23.94 ± 17.28	2.18	0.117
*γ*-GGT (IU/L)	14.60 (11.85–20.75)	21.70 (15.55–38.68)^a^	25.55 (13.08–48.58)	2.85	0.061
UA (mmol/L)	255.00 (233.80–286.65)	273.90 (229.55–328.10)	360.00 (323.55–459.25)^b,d^	22.52	<0.001
Cre (mmol/L)	53.30 (50.05–59.05)	51.05 (46.53–59.75)	59.90 (55.25–63.63)	0.58	0.561
BUN (mmol/L)	6.51 ± 10.69	4.97 ± 1.82	4.64 ± 1.30	1.03	0.36
WBC (10^9^/L)	5.76 ± 1.60	6.10 ± 1.70	6.80 ± 1.58^b,c^	7.15	<0.001
CRP (mg/L)	N	5.41 ± 1.44	3.93 ± 2.33^c^	2.89	0.005
Testosterone (nmol/L)	1.43 ± 1.38	N	2.04 ± 1.56^b^	−5.42	<0.001
FSH (mIU/L)	4.72 ± 4.79	N	4.06 ± 1.49	1.34	0.184
LH (mIU/L)	8.99 ± 8.08	N	8.92 ± 5.15	0.09	0.932
LH/FSH	1.63 (0.87–2.16)	N	2.02 (1.37–2.65)^a^	−2.10	0.036
E2 (pg/mL)	110.55 ± 123.39	N	67.50 ± 51.19^b^	3.29	0.002
P (ng/mL)	4.10 ± 7.50	N	1.52 ± 3.16^b^	3.25	0.003
PRL (ng/mL)	17.98 ± 10.95	N	18.17 ± 11.90	−0.16	0.872

Notes: Data are expressed as mean ± SD or median and interquartile range (25–75%). *P* values are from ANOVA, unpaired *T* test or Mann-Whitney *U* test. *N* means not available. *n* = 32–66 healthy subjects, 47–53 T2DM subjects, 31–41 PCOS subjects. ^a^ *P* < 0.05 and ^b^ *P* < 0.01 compared with the healthy group. ^c^ *P* < 0.05 and ^d^ *P* < 0.01 compared with the T2DM group. BMI: body mass index; WHR: waist-to-hip ratio; SBP: systolic blood pressure; DBP: diastolic blood pressure; WBC: white blood cell count; CRP: C-reactive protein; FBG: fasting blood glucose; FINS: fasting insulin; HbA1c: hemoglobin A1c; HOMA-IR: HOMA-*β*, homoeostasis model assessment of insulin resistance and insulin secretion; TC: total cholesterol; TG: triglycerides; LDL-C: low-density lipoprotein cholesterol; HDL-C: high-density lipoprotein cholesterol; AST: aspartate transaminase; ALT: alanine transaminase; *γ*-GGT: gamma-glutamyl transpeptidase; UA: uric acid; Cre: creatinine; BUN: blood urea nitrogen; T2DM: type 2 diabetes mellitus; PCOS: polycystic ovary syndrome; FSH: follicle-stimulating hormone; LH: luteinizing hormone; E2: estradiol; P: progesterone; PRL: prolactin.

**Table 2:** Metabolic characteristics of T2DM and PCOS females in normal-weight and overweight subgroups categorized by BMI.

	T2DM normal-weight	T2DM overweight/obese	*P*	PCOS normal-weight	PCOS overweight/obese	*P*
Age (years)	47.48 ± 4.86	46.72 ± 5.01	0.589	20.33 ± 5.18	23.66 ± 5.65	0.087
BMI (kg/m^2^)	21.90 ± 1.64	27.00 ± 2.46	<0.001	22.24 ± 1.44	28.97 ± 3.37	<0.001
WHR	0.83 ± 0.10	0.93 ± 0.07	<0.001	0.86 ± 0.07	0.91 ± 0.10	0.064
SBP (mmHg)	122.76 ± 12.53	126.56 ± 10.03	0.227	115.27 ± 11.68	119.21 ± 10.60	0.316
DBP (mmHg)	76.48 ± 9.33	77.34 ± 8.54	0.729	71.73 ± 6.05	77.79 ± 8.66	0.040
FBG (mmol/L)	7.10 ± 1.99	7.84 ± 3.46	0.385	4.27 ± 0.62	4.93 ± 2.60	0.391
FINS (mU/mL)	9.08 ± 5.15	11.29 ± 6.88	0.216	11.63 ± 3.36	15.85 ± 6.37	0.037
HbA1c (%)	8.42 ± 2.72	9.02 ± 2.23	0.405	5.20 ± 0.25	6.41 ± 1.87	0.003
HOMA-IR	2.15 (1.26–3.17)	3.14 (2.07–5.28)	0.106	1.61 (1.02–2.01)	3.19 (2.63–4.23)	0.007
HOMA-*β*	44.05 (21.23–98.67)	56.66 (30.75–87.21)	0.611	259.20 (181.13–368.19)	158.44 (133.33–474.03)	0.915
OGTT-30 min insulin (mU/mL)	23.80 (11.05–30.05)	17.90 (13.3–28.1)	0.960	40.55 (35.43–77.10)	62.10 (36.40–98.00)	0.231
OGTT-60 min insulin (mU/mL)	21.50 (10.35–53.70)	28.00 (20.90–55.35)	0.490	43.90 (40.75–52.00)	91.40 (60.70–138.00)	0.004
OGTT-120 min insulin (mU/mL)	26.00 (14.15–48.30)	30.60 (18.45–59.05)	0.375	34.30 (24.28–64.35)	84.60 (57.60–179.10)	0.034
OGTT-180 min insulin (mU/mL)	13.50 (10.20–24.10)	22.90 (13.25–35.40)	0.043	14.05 (5.98–27.30)	55.70 (34.30–88.90)	0.601
OGTT-IAUC	3886.50 (2090.25–7894.50)	4779.00 (3212.25–7833.00)	0.397	6000.75 (5749.13–8155.88)	15915.00 (9030.00–21195.00)	0.041
TG (mmol/L)	1.83 ± 1.13	2.05 ± 1.24	0.513	1.43 ± 0.74	1.69 ± 0.91	0.444
TC (mmol/L)	4.66 ± 0.83	4.79 ± 1.02	0.632	3.86 ± 0.64	4.67 ± 1.09	0.045
LDL-C (mmol/L)	3.08 ± 0.66	3.25 ± 0.86	0.464	2.44 ± 0.60	3.01 ± 0.81	0.065
HDL-C (mmol/L)	1.25 ± 0.33	1.12 ± 0.22	0.111	1.14 ± 0.14	1.23 ± 0.44	0.535
AST (IU/L)	19.68 ± 10.85	23.50 ± 15.66	0.359	17.42 ± 4.78	24.98 ± 12.71	0.094
ALT (IU/L)	23.18 ± 13.93	27.00 ± 20.49	0.483	14.15 ± 7.08	28.25 ± 18.74	0.002
*γ*-GGT (IU/L)	16.10 (13.70–32.70)	21.70 (15.50–34.10)	0.062	17.25 (12.03–29.98)	29.50 (23.90–56.70)	0.018
WBC (10^9^/L)	5.67 ± 1.42	6.36 ± 2.10	0.182	6.43 ± 1.93	7.25 ± 1.53	0.144
CRP (mg/L)	5.22 ± 0.60	5.53 ± 1.79	0.155	4.71 ± 2.16	4.10 ± 1.90	0.423

Notes: Data are presented as mean ± standard deviation for normally distributed variables and median (interquartile range) for nonnormally distributed variables. *P* values are from unpaired *T* test or Mann-Whitney *U* test. *n* = 14–21 normal-weight T2DM subjects, *n* = 19–32 overweight/obese T2DM subjects, *n* = 7–12 normal-weight PCOS subjects, *n* = 19–29 overweight/obese PCOS subjects. OGTT: oral glucose tolerance test; IAUC: insulin area under the curve.

**Table 3:** The correlations between plasma asprosin level and metabolic risk factors in all subjects.

	Total		Total (age-adjusted)	
	*r*	*P*	*r*	*P*
Age (years)	0.085	0.288	—	—
BMI (kg/m^2^)	0.215	0.006	0.169	0.033
WHR	0.312	<0.001	0.229	0.004
SBP (mmHg)	0.203	0.010	0.212	0.008
DBP (mmHg)	0.23	0.004	0.175	0.028
FBG (mmol/L)	0.332	<0.001	0.579	<0.001
FINS (mU/mL)	0.194	0.014	0.198	0.013
HbA1c (%)	0.581	<0.001	0.568	<0.001
HOMA-IR	0.363	<0.001	0.46	<0.001
HOMA-*β*	−0.291	<0.001	−0.024	0.776
TG (mmol/L)	0.361	<0.001	0.324	<0.001
TC (mmol/L)	0.242	0.002	0.235	0.003
LDL-C (mmol/L)	0.263	0.001	0.230	0.005
HDL-C (mmol/L)	−0.202	0.013	−0.114	0.165
AST (IU/L)	−0.011	0.890	0.009	0.914
ALT (IU/L)	0.034	0.677	0.047	0.568
*γ*-GGT (IU/L)	0.263	0.001	0.020	0.809
UA (mmol/L)	0.184	0.028	0.198	0.018
Cre (mmol/L)	−0.019	0.820	−0.061	0.454
BUN (mmol/L)	0.073	0.373	−0.003	0.972
WBC (10^9^/L)	0.153	0.059	0.173	0.033

Notes: Correlations between variables were analyzed by Spearman analysis or an age-adjusted partial correlation test.

**Table 4:** The correlations between plasma asprosin level and metabolic risk factors in the T2DM group and BMI-categorized subgroups of T2DM.

	T2DM		T2DM (age-adjusted)		T2DM normal-weight(age-adjusted)		T2DM overweight/obese (age-adjusted)	
	*r*	*P*	*r*	*P*	*r*	*P*	*r*	*P*
BMI (kg/m^2^)	0.243	0.079	−0.104	0.464	−0.293	0.209	0.119	0.523
WHR	−0.058	0.683	−0.255	0.070	−0.215	0.377	−0.480	0.006
SBP (mmHg)	0.170	0.225	0.200	0.156	0.505	0.023	−0.007	0.971
DBP (mmHg)	0.097	0.492	0.037	0.793	0.405	0.077	−0.142	0.448
FBG (mmol/L)	0.366	0.007	0.536	<0.001	0.473	0.035	0.539	0.002
FINS (mU/mL)	−0.100	0.476	0.072	0.61	0.408	0.074	−0.099	0.596
OGTT-120 min insulin (mU/mL)	−0.283	0.073	−0.275	0.086	−0.519	0.048	−0.219	0.305
OGTT-IAUC	−0.335	0.057	−0.249	0.170	−0.534	0.060	−0.190	0.450
HbA1c (%)	0.322	0.024	0.405	0.004	0.469	0.043	0.378	0.047
HOMA-IR	0.136	0.330	0.392	0.004	0.526	0.017	0.288	0.116
HOMA-*β*	−0.382	0.005	−0.039	0.781	−0.185	0.434	−0.041	0.826
TG (mmol/L)	0.194	0.169	0.190	0.181	−0.056	0.819	0.279	0.129
TC (mmol/L)	0.049	0.731	0.006	0.966	0.107	0.664	−0.019	0.919
LDL-C (mmol/L)	0.002	0.992	−0.038	0.797	0.055	0.829	−0.067	0.726
HDL-C (mmol/L)	−0.055	0.706	−0.08	0.583	0.434	0.072	−0.395	0.031
UA (mmol/L)	0.112	0.454	0.080	0.595	−0.032	0.900	0.002	0.991
WBC (10^9^/L)	0.294	0.041	0.179	0.222	0.231	0.371	0.271	0.250
CRP (mg/L)	0.338	0.038	0.265	0.113	0.311	0.279	0.284	0.200

Notes: Correlations between variables were analyzed by an age-adjusted partial correlation test.

**Table 5:** Association of plasma asprosin with T2DM or PCOS in fully adjusted models.

Model adjustment	T2DM		PCOS	
	OR, 95% CI	*P*	OR, 95% CI	*P*
Age	2.092 (1.446–3.028)	<0.001	1.670 (1.104–2.526)	0.015
Age, BMI	2.183 (1.470–3.243)	<0.001	1.774 (1.068–2.949)	0.027
Age, BMI, WBC	2.242 (1.480–3.398)	<0.001	1.979 (1.126–3.476)	0.018
Age, BMI, SBP, DSP	2.288 (1.483–3.527)	<0.001	1.843 (1.095–3.104)	0.021
Age, BMI, WBC, SBP, DSP	2.253 (1.440–3.523)	<0.001	2.156 (1.160–4.005)	0.015
Age, BMI, WBC, SBP, DSP, HDL, TG, LDL, TC	2.149 (1.307–3.531)	0.003	2.483 (1.102–5.596)	0.028
Age, BMI, WBC, SBP, DSP, HDL, TG, LDL, TC, ALT, AST	2.789 (1.362–5.748)	0.005	2.385 (0.980–5.809)	0.056
Age, BMI, WBC, SBP, DSP, HDL, TG, LDL, TC, ALT, AST, *γ*-GGT	2.790 (1.132–6.874)	0.026	2.321 (0.917–5.874)	0.075

Note: Results of binary logistic regression analyses are presented. OR: odds ratio; CI: confidence interval.

**Table 6:** The relationships between plasma asprosin level and metabolic and sex hormone profiles of PCOS females and the subgroups categorized by BMI.

	PCOS		PCOS (age-adjusted)		PCOS normal-weight(age-adjusted)		PCOS overweight/obese (age-adjusted)	
	*r*	*P*	*r*	*P*	*r*	*P*	*r*	*P*
Age (years)	−0.118	0.463	—	—	—	—	—	—
BMI (kg/m^2^)	0.201	0.207	0.150	0.365	0.276	0.411	−0.164	0.403
WHR	−0.087	0.590	0.083	0.609	−0.090	0.793	0.014	0.946
FBG (mmol/L)	0.148	0.355	0.636	0.000	−0.019	0.956	0.692	<0.001
FINS (mU/mL)	0.328	0.036	0.179	0.269	−0.079	0.818	0.090	0.649
OGTT-30 min insulin (mU/mL)	0.188	0.357	0.169	0.419	0.664	0.150	−0.016	0.949
EHCT-0 min-insulin (mU/mL)	0.699	0.002	0.456	0.076	0.541	0.636	0.365	0.244
EHCT-90 min-insulin (mU/mL)	0.429	0.086	0.311	0.240	−0.822	0.385	0.249	0.435
EHCT-180 min-insulin (mU/mL)	0.544	0.024	0.396	0.129	−0.344	0.776	0.385	0.216
HOMA-IR	0.429	0.005	0.544	0.000	−0.109	0.749	0.563	0.002
HOMA-*β*	−0.054	0.773	−0.026	0.875	−0.064	0.880	−0.229	0.319
HbA1c (%)	0.630	0.000	0.645	0.000	0.593	0.092	0.602	0.001
TG (mmol/L)	0.250	0.135	0.206	0.229	−0.315	0.447	0.233	0.242
TC (mmol/L)	0.226	0.178	0.389	0.019	0.322	0.422	0.327	0.096
LDL-c (mmol/L)	0.400	0.019	0.466	0.006	0.331	0.424	0.421	0.041
HDL-c (mmol/L)	−0.284	0.093	0.163	0.349	0.354	0.390	0.149	0.466
APOE (mg/dL)	0.310	0.070	0.503	0.002	0.521	0.150	0.464	0.022
APOB (g/L)	0.411	0.013	0.439	0.008	0.086	0.827	0.400	0.047
APOA1 (g/L)	−0.279	0.100	0.062	0.722	−0.317	0.406	0.078	0.711
T (nmol/L)	0.294	0.062	0.340	0.027	−0.118	0.729	0.379	0.047
FSH (mIU/L)	0.153	0.340	0.092	0.573	0.504	0.114	0.119	0.547
LH (mIU/L)	0.068	0.674	0.022	0.892	0.348	0.295	0.031	0.977
LH/FSH	−0.080	0.621	−0.063	0.698	−0.091	0.790	−0.077	0.697
E2 (pg/mL)	−0.091	0.570	−0.132	0.419	0.669	0.024	−0.304	0.116
P (ng/mL)	−0.195	0.221	−0.232	0.150	−0.271	0.420	−0.331	0.085
PRL (ng/mL)	−0.414	0.007	−0.39	0.013	−0.293	0.382	−0.464	0.013
SHBG (nmol/L)	−0.429	0.016	−0.247	0.188	−0.924	0.003	−0.115	0.610
DHEA-S (*μ*g/dl)	0.042	0.833	0.166	0.407	0.836	0.078	0.379	0.090
17*α*-OHP (ng/mL)	0.044	0.853	0.011	0.966	0.855	0.145	0.018	0.952
AMH (ng/mL)	−0.202	0.407	−0.201	0.423	−0.938	0.062	−0.085	0,783
WBC (10^9^/L)	0.112	0.504	0.102	0.546	0.050	0.899	0.026	0.897
CRP (mg/L)	−0.009	0.958	−0.002	0.989	0.209	0.619	0.124	0.547
TNF-*α* (pg/mL)	0.236	0.266	0.107	0.628	0.962	0.176	0.003	0.989
IL-6 (pg/mL)	0.449^∗^	0.028	0.229	0.293	−0.474	0.686	0.174	0.477
IL-8 (pg/mL)	0.342	0.120	0.124	0.592	−0.447	0.705	0.052	0.481

Note: Correlations between variables were analyzed by Spearman's correlation test and an age-adjusted partial correlation test. EHCT: euglycemic-hyperinsulinemic clamp test; SHBG: sex hormone-binding globulin; DHEA-S: dehydroepiandrosterone sulfate; 17*α*-OHP: 17-*α*-hydroxyprogesterone; AMH: anti-Mullerian hormone; TNF-*α*: tumor necrosis factor-*α*; IL-6: interleukin-6.

## Data Availability

The clinical data used to support the findings of this study are restricted by the Ethics Committee of Xinqiao Hospital in order to protect patient privacy. Data are available from Chinese Clinical Trial Registry (number: ChiCTR-ROC-17010719) for researchers who meet the criteria for access to confidential data. The statistical data used to support the findings of this study are available from the corresponding author upon request.

## Supplementary Materials

Supplementary Materials Supplementary Table 1: medication history profile by group. Supplementary Table 2: clinical, metabolic, and sex hormone features of healthy, T2DM, and PCOS female subjects. Supplementary Table 3: metabolic characteristics of T2DM and PCOS females in normal-weight and overweight subgroups categorized by BMI. Supplementary Table 4: correlations between plasma asprosin level and metabolic risk factors in all subjects. Supplementary Table 5: correlations between plasma asprosin level and metabolic risk factors in the T2DM group and BMI-categorized subgroups of T2DM. Supplementary Table 6: relationships between plasma asprosin level and metabolic and sex hormone profiles of PCOS females and subgroups categorized by BMI. Supplementary Table 7: association of plasma asprosin with T2DM or PCOS in fully adjusted models. Supplementary Figure 1: plasma asprosin concentrations in groups and the overweight/obese subgroups.
