# Supplementary material for: Plasma Asprosin Levels Are Associated with Glucose Metabolism, Lipid, and Sex Hormone Profiles in Females with Metabolic-Related Diseases
Source: Mediators Inflamm. 2018 Nov 6;2018:7375294. doi: 10.1155/2018/7375294 (PMC6247534; doi:10.1155/2018/7375294)
Supplement: Supplementary Materials — Supplementary Table 1: medication history profile by group. Supplementary Table 2: clinical, metabolic, and sex hormone features of healthy, T2DM, and PCOS female subjects. Supplementary Table 3: metabolic characteristics of T2DM and PCOS females in normal-weight and overweight subgroups categorized by BMI. Supplementary Table 4: correlations between plasma asprosin level and metabolic risk factors in all subjects. Supplementary Table 5: correlations between plasma asprosin level and metabolic risk factors in the T2DM group and BMI-categorized subgroups of T2DM. Supplementary Table 6: relationships between plasma asprosin level and metabolic and sex hormone profiles of PCOS females and subgroups categorized by BMI. Supplementary Table 7: association of plasma asprosin with T2DM or PCOS in fully adjusted models. Supplementary Figure 1: plasma asprosin concentrations in groups and the overweight/obese subgroups. [file 7375294.f1.docx]

Supplementary Table 1: Medication history profile by group.

| Drug | | Number/Percentage (%) | | |
| --- | --- | --- | --- | --- |
|  |  | Healthy  (n=66) | T2DM  (n=53) | PCOS  (n=41) |
| Anti-diabetic drugs | Metformin | 0/0 | 0/0 | 3/7.32 |
| Anti-hypertensive drug | Angiotensin-converting enzyme inhibitor (ACEI) | 0/0 | 1/1.89 | 0/0 |
|  | Angiotensin receptor blocker (ARB) | 0/0 | 5/9.43 | 0/0 |
|  | Adrenergic β receptor blockers | 0/0 | 4/7.55 | 1/2.44 |
|  | Calcium channel blocker | 0/0 | 3/5.66 | 0/0 |
|  | Diuretic | 0/0 | 3/5.66 | 0/0 |
|  | Nitroglycerin | 0/0 | 1/1.89 | 0/0 |
| Anti-hyperlipidemia drugs | Statin | 0/0 | 6/11.32 | 1/2.44 |
| Total | Single/combined drugs | 0/0 | 11/20.75 | 3/7.32 |

Supplementary Table 2: Clinical, metabolic, and sex hormone features of healthy, T2DM, and PCOS female subjects.

|  | Healthy | T2DM | PCOS | F/t/Z | P |
| --- | --- | --- | --- | --- | --- |
| Age (years) | 37.02±8.16 | 47.07±4.68^b^ | 22.39±5.73^b, d^ | 135.97 | <0.001 |
| BMI (kg/m^2^) | 22.68±4.00 | 25.06±3.45 | 26.77±4.32^b^ | 13.80 | <0.001 |
| WHR | 0.73±0.04 | 0.89±0.09^b^ | 0.89±0.09^b^ | 94.18 | <0.001 |
| SBP (mmHg) | 116.05±9.57 | 125.62±11.38^b^ | 117.25±10.67^d^ | 11.67 | <0.001 |
| DBP (mmHg) | 73.09±7.82 | 76.29±7.70^a^ | 75.11±7.79 | 2.30 | 0.104 |
| FBG (mmol/L) | 4.75±0.55 | 7.95±3.09^b^ | 4.72±2.30^d^ | 36.31 | <0.001 |
| FINS (mU/mL) | 7.85±5.94 | 10.36±5.52^a^ | 14.66±6.15 ^b, d^ | 16.15 | <0.001 |
| HbA1c (%) | 5.51±0.31 | 8.85±2.51^b^ | 5.81±1.14^d^ | 48.95 | <0.001 |
| HOMA-IR | 1.68±1.37 | 3.80±2.70^b^ | 3.02±1.61^d^ | 16.95 | <0.001 |
| HOMA-β | 112.12  (78.49-157.88) | 42.12 (23.49-66.48) ^a^ | 318.39 (161.27-440.14) ^b, d^ | 32.10 | 0.001 |
| TC (mmol/L) | 4.18±0.76 | 4.71±0.98^b^ | 4.43±1.08 | 4.27 | 0.016 |
| TG (mmol/L) | 0.94 (0.73-1.25) | 1.71(1.30-3.11) b | 1.01 (0.67-1.51) ^b^ | 9.46 | <0.001 |
| LDL-C (mmol/L) | 2.58±0.64 | 3.14±0.81^b^ | 2.79±0.79^c^ | 7.55 | <0.001 |
| HDL-C (mmol/L) | 1.43±0.33 | 1.16±0.29^b^ | 1.23±0.40^b^ | 8.91 | <0.001 |
| AST (IU/L) | 22.83±16.16 | 21.32±13.96 | 20.92±8.46 | 0.25 | 0.780 |
| ALT (IU/L) | 18.64±18.74 | 15.50  (12.55-32.80) | 15.40  (12.25-29.05) | 1.59 | 0.208 |
| γ-GGT (IU/L) | 14.60  (11.85-20.75) | 19.50  (14.20-35.00) ^a^ | 19.40  (12.35-32.80) | 2.31 | 0.103 |
| UA (mmol/L) | 255.00  (233.80-286.65) | 274.30  (239.15-353.65) | 352.30  (309.20-439.65) ^b, d^ | 18.42 | <0.001 |
| Cre (mmol/L) | 53.30  (50.05-59.05) | 51.60 (44.45-59.30) | 59.90  (54.25-64.85) ^b, d^ | 7.32 | 0.001 |
| BUN (mmol/L) | 6.51±10.69 | 4.78±1.67 | 4.74±1.30 | 0.93 | 0.399 |
| WBC (10^9^/L) | 5.76±1.60 | 5.95±1.77 | 7.04±1.66^b, d^ | 7.16 | 0.001 |
| CRP (mg/L) | N | 5.34±1.49 | 4.32±2.01^c^ | 2.31 | 0.024 |
| Testosterone (nmol/L) | 1.43±1.38 | N | 2.09±1.60 | -5.28 | <0.001 |
| FSH (mIU/L) | 4.72±4.79 | N | 4.19±1.44 | 1.18 | 0.243 |
| LH (mIU/L) | 8.99±8.08 | N | 9.22±5.19 | -0.07 | 0.945 |
| LH/FSH | 1.63 (0.87-2.16) | N | 2.04 (1.36-2.66) | -1.95 | 0.052 |
| E2 (pg/ml) | 110.55±123.39 | N | 66.70±51.23 | 3.31 | 0.002 |
| P (ng/ml) | 4.10±7.50 | N | 1.42±3.12 | 3.29 | 0.002 |
| PRL (ng/ml) | 17.98±10.95 | N | 18.42±12.28 | -0.25 | 0.804 |

**Notes:** Data are expressed as the mean ± SD or median and interquartile range (25–75%). P values are from ANOVA, unpaired t test or Mann-Whitney U test. N means not available. ^a^ 𝑃< 0.05; ^b^ 𝑃< 0.01 compared with the healthy group. ^c^ 𝑃< 0.05; ^d^ 𝑃< 0.01 compared with the T2DM group. Data were from participants without medication history of anti-diabetic, anti-hypertensive or anti-hyperlipidemia drugs.

**Abbreviations:** BMI, body mass index; WHR, waist-to-hip ratio; SBP, systolic blood pressure; DBP, diastolic blood pressure; WBC, white blood cell count; CRP, C-reactive protein; FBG, fasting blood glucose; FINS, fasting insulin; HbA1c, hemoglobin A1c; HOMA-IR, HOMA-β, homoeostasis model assessment of insulin resistance and insulin secretion; TC, total cholesterol; TG, triglycerides; LDL-C, low-density lipoprotein cholesterol; HDL-C, high-density lipoprotein cholesterol; AST, aspartate transaminase; ALT, alanine transaminase; 𝛾-GGT, gamma-glutamyl transpeptidase; UA, uric acid; Cre, creatinine; BUN, blood urea nitrogen; T2DM, type 2 diabetes mellitus; PCOS, polycystic ovary syndrome; FSH, follicle-stimulating hormone; LH, luteinizing hormone; E2, estradiol; P, progesterone; PRL, prolactin.

Supplementary Table 3: Metabolic characteristics of T2DM and PCOS females in normal-weight and overweight subgroups categorized by BMI.

|  | T2DM  Normal-  weight | T2DM Overweight/  obese | P | PCOS  Normal-  weight | PCOS Overweight/  obese | P |
| --- | --- | --- | --- | --- | --- | --- |
| Age (years) | 48.20±3.55 | 46.44±5.16 | 0.249 | 20.33±5.18 | 23.35±5.81 | 0.134 |
| BMI (kg/m^2^) | 21.47±1.71 | 27.05±2.39 | <0.001 | 22.24±1.44 | 28.87±3.51 | <0.001 |
| WHR | 0.83±0.10 | 0.92±0.07 | <0.001 | 0.86±0.07 | 0.91±0.09 | 0.095 |
| SBP (mmHg) | 123.00±12.72 | 127.07±10.53 | 0.271 | 115.27±11.68 | 118.12±10.32 | 0.469 |
| DBP (mmHg) | 74.40±6.35 | 77.33±8.28 | 0.241 | 71.73±6.05 | 76.54±8.10 | 0.086 |
| FBG (mmol/L) | 7.26±2.06 | 8.33±3.52 | 0.287 | 4.27±0.62 | 4.92±2.74 | 0.426 |
| FINS (mU/mL) | 9.79±5.76 | 10.68±5.47 | 0.622 | 11.63±3.36 | 16.05±6.68 | 0.038 |
| HbA1c (%) | 8.32±2.76 | 9.18±2.35 | 0.307 | 5.20±0.25 | 6.07±1.27 | 0.041 |
| HOMA-IR | 2.16  (1.16-3.67) | 2.50  (2.05-4.90) | 0.265 | 1.61  (1.02-2.01) | 3.19  (2.51-4.57) | 0.013 |
| HOMA-β | 45.67  (20.54-104.91) | 43.56  (29.83-73.27) | 0.927 | 259.20 (181.13-368.19) | 383.56 (132.15-602.59) | 0.902 |
| OGTT-30 min insulin (mU/mL) | 19.70  (13.70-29.50) | 17.1  (11.80-25.30) | 0.747 | 40.55  (35.43-77.10) | 90.60  (66.45-116.25) | 0.127 |
| OGTT-60 min insulin (mU/mL) | 26.70  (9.60-52.35) | 25.30  (19-32.30) | 0.885 | 43.90  (40.75-52.00) | 91.40  (61.95-156.60) | 0.002 |
| OGTT-120 min insulin (mU/mL) | 23.85  (8.20-43.85) | 27.80  (18.40-46.10) | 0.314 | 34.30  (24.28-64.35) | 84.60  (59.10-181.25) | 0.040 |
| OGTT-180 min insulin (mU/mL) | 12.70  (8.93-18.53) | 21  (13-29.10) | 0.062 | 14.05  (5.98-27.30) | 55.70  (40.40-86.70) | 0.657 |
| OGTT-IAUC | 3915.00 (1657.13-7220.25) | 4180.50 (3087-6232.50) | 0.605 | 6000.75 (5749.13-8155.88) | 15915 (9386.25-23112.75) | 0.033 |
| TG (mmol/L) | 1.76±1.20 | 1.97±0.95 | 0.541 | 1.43±0.74 | 1.62±0.93 | 0.580 |
| TC (mmol/L) | 4.54±0.72 | 4.80±1.10 | 0.421 | 3.86±0.64 | 4.63±1.14 | 0.068 |
| LDL-C (mmol/L) | 2.94±0.49 | 3.25±0.93 | 0.253 | 2.44±0.60 | 2.94±0.82 | 0.112 |
| HDL-C (mmol/L) | 1.24±0.38 | 1.12±0.22 | 0.282 | 1.14±0.14 | 1.26±0.47 | 0.260 |
| AST (IU/L) | 16.20±5.05 | 24.30±16.55 | 0.034 | 17.42±4.78 | 22.35±9.28 | 0.143 |
| ALT (IU/L) | 21.07±13.88 | 27.71±20.75 | 0.284 | 14.15±7.08 | 24.91±16.31 | 0.046 |
| γ-GGT (IU/L) | 15.5  (13.00-28.95) | 21.7  (15.60-32.10) | 0.002 | 17.25  (12.03-29.98) | 29.50  (21.95-55.85) | 0.036 |
| WBC (10^9^/L) | 5.50±1.49 | 6.19±1.89 | 0.240 | 6.43±1.93 | 7.31±1.54 | 0.125 |
| CRP (mg/L) | 5.11±0.38 | 5.48±1.88 | 0.502 | 4.71±2.16 | 4.18±1.98 | 0.505 |

**Notes:** Data are presented as the mean ± standard deviation for normally distributed variables and median (interquartile range) for nonnormally distributed variables. P values are from unpaired t test or Mann-Whitney U test. Data were from participants without medication history of anti-diabetic, anti-hypertensive or anti-hyperlipidemia drugs.

**Abbreviations:** OGTT, oral glucose tolerance test; IAUC, insulin area under curve.

Supplementary Table 4: Correlations between plasma asprosin level and metabolic risk factors in all subjects.

|  | Total | | Total  (Age-adjusted) | |
| --- | --- | --- | --- | --- |
|  | r | P | r | P |
| Age (years) | 0.048 | 0.563 | - | - |
| BMI (kg/m^2^) | 0.200 | 0.015 | 0.143 | 0.086 |
| WHR | 0.305 | <0.001 | 0.195 | 0.051 |
| SBP (mmHg) | 0.216 | 0.009 | 0.228 | 0.006 |
| DBP (mmHg) | 0.221 | 0.007 | 0.164 | 0.049 |
| FBG (mmol/L) | 0.335 | <0.001 | 0.612 | <0.001 |
| FINS (mU/mL) | 0.234 | 0.004 | 0.216 | 0.009 |
| HbA1c (%) | 0.585 | <0.001 | 0.607 | <0.001 |
| HOMA-IR | 0.384 | <0.001 | 0.488 | <0.001 |
| HOMA-β | -0.293 | <0.001 | -0.092 | 0.297 |
| TG (mmol/L) | 0.324 | <0.001 | 0.303 | <0.001 |
| TC (mmol/L) | 0.211 | 0.012 | 0.207 | 0.014 |
| LDL-C (mmol/L) | 0.238 | 0.005 | 0.203 | 0.018 |
| HDL-C (mmol/L) | -0.194 | 0.022 | -0.100 | 0.245 |
| AST (IU/L) | -0.052 | 0.551 | -0.003 | 0.973 |
| ALT (IU/L) | 0.013 | 0.876 | 0.020 | 0.817 |
| γ-GGT (IU/L) | 0.257 | 0.003 | 0.024 | 0.787 |
| UA (mmol/L) | 0.148 | 0.091 | 0.162 | 0.064 |
| Cre (mmol/L) | -0.001 | 0.989 | 0.002 | 0.984 |
| BUN (mmol/L) | 0.126 | 0.144 | 0.011 | 0.898 |
| WBC (10^9^/L) | 0.159 | 0.060 | 0.170 | 0.044 |

**Notes:** Correlations between variables were analyzed by Spearman analysis or an age-adjusted partial correlation test. Data were from participants without medication history of anti-diabetic, anti-hypertensive or anti-hyperlipidemia drugs.

Supplementary Table 5: Correlations between plasma asprosin level and metabolic risk factors in the T2DM group and BMI-categorized subgroups of T2DM.

|  | T2DM | | T2DM (Age-adjusted) | | T2DM  Normal-weight (Age-adjusted) | | T2DM  Overweight/obese (Age-adjusted) | |
| --- | --- | --- | --- | --- | --- | --- | --- | --- |
|  | r | P | r | P | r | P | r | P |
| BMI (kg/m^2^) | 0.167 | 0.291 | 0.061 | 0.706 | -0.391 | 0.167 | 0.043 | 0.834 |
| WHR | -0.222 | 0.162 | -0.407 | 0.009 | -0.400 | 0.176 | -0.652 | <0.001 |
| SBP (mmHg) | 0.189 | 0.231 | 0.233 | 0.142 | 0.388 | 0.170 | 0.082 | 0.692 |
| DBP (mmHg) | 0.061 | 0.702 | 0.002 | 0.992 | 0.138 | 0.639 | -0.101 | 0.623 |
| FBG (mmol/L) | 0.425 | 0.005 | 0.573 | <0.001 | 0.356 | 0.211 | 0.604 | 0.001 |
| FINS (mU/mL) | -0.070 | 0.661 | 0.119 | 0.460 | 0.557 | 0.038 | -0.110 | 0.592 |
| OGTT-120 min insulin (mU/mL) | -.0348 | 0.040 | -0.303 | 0.082 | -0.668 | 0.025 | -0.271 | 0.222 |
| OGTT-IAUC | -0.412 | 0.029 | -0.278 | 0.016 | -0.697 | 0.025 | -0.234 | 0.382 |
| HbA1c (%) | 0.357 | 0.026 | 0.445 | 0.005 | 0.503 | 0.067 | 0.489 | 0.018 |
| HOMA-IR | 0.161 | 0.308 | 0.445 | 0.005 | 0.593 | 0.026 | 0.329 | 0.101 |
| HOMA-β | -0.429 | 0.005 | -0.245 | 0.122 | -0.055 | 0.852 | -0.401 | 0.042 |
| TG (mmol/L) | 0.062 | 0.699 | 0.150 | 0.350 | -0.309 | 0.282 | 0.360 | 0.070 |
| TC (mmol/L) | -0.057 | 0.722 | -0.065 | 0.686 | -0.212 | 0.467 | -0.064 | 0.755 |
| LDL-C (mmol/L) | -0.079 | 0.630 | -0.095 | 0.566 | -0.512 | 0.074 | -0.100 | 0.636 |
| HDL-C (mmol/L) | -0.065 | 0.692 | -0.081 | 0.624 | 0.456 | 0.117 | -0.467 | 0.019 |
| UA (mmol/L) | -0.087 | 0.602 | -0.020 | 0.907 | -0.228 | 0.434 | -0.098 | 0.663 |
| WBC (10^9^/L) | 0.329 | 0.038 | 0.194 | 0.236 | 0.239 | 0.431 | 0.253 | 0.222 |
| CRP (mg/L) | 0.187 | 0.315 | 0.242 | 0.197 | -0.048 | 0.889 | 0.295 | 0.235 |

**Notes:** Correlations between variables were analyzed by an age-adjusted partial correlation test. Data were from participants without medication history of anti-diabetic, anti-hypertensive or anti-hyperlipidemia drugs.

Supplementary Table 6: Relationships between plasma asprosin level and metabolic and sex hormone profiles of PCOS females and subgroups categorized by BMI.

|  | PCOS | | PCOS  (Age-adjusted) | | PCOS  Normal-weight (Age-adjusted) | | PCOS  Overweight/obese  (Age-adjusted) | |  |
| --- | --- | --- | --- | --- | --- | --- | --- | --- | --- |
|  |  |  |  |  |  |  |  |  |  |
|  | r | P | r | P | r | P | r | P | |
| Age (years) | -0.089 | 0.594 |  |  | - | - | - | - | |
| BMI (kg/m^2^) | 0.214 | 0.198 | 0.134 | 0.430 | 0.276 | 0.411 | -0.197 | 0.346 | |
| WHR | -0.074 | 0.658 | 0.116 | 0.494 | -0.090 | 0.793 | 0.065 | 0.757 | |
| FBG (mmol/L) | 0.189 | 0.257 | 0.668 | <0.001 | -0.019 | 0.956 | 0.733 | <0.001 | |
| FINS (mU/mL) | 0.320 | 0.050 | 0.168 | 0.322 | -0.079 | 0.818 | 0.068 | 0.746 | |
| OGTT-30 min insulin (mU/mL) | 0.153 | 0.476 | 0.141 | 0.521 | 0.664 | 0.150 | -0.105 | 0.669 | |
| EHCT-0 min-insulin (mU/mL) | 0.705 | 0.002 | 0.425 | 0.115 | 0.541 | 0.636 | 0.304 | 0.363 | |
| EHCT-90 min-insulin (mU/mL) | 0.397 | 0.128 | 0.261 | 0.347 | -0.822 | 0.385 | 0.141 | 0.679 | |
| EHCT-180 min-insulin (mU/mL) | 0.494 | 0.052 | 0.308 | 0.264 | -0.344 | 0.776 | 0.252 | 0.456 | |
| HOMA-IR | 0.443 | 0.005 | 0.566 | <0.001 | -0.109 | 0.749 | 0.578 | 0.002 | |
| HOMA-β | -0.118 | 0.550 | -0.15 | 0.454 | -0.064 | 0.880 | -0.282 | 0.257 | |
| HbA1c (%) | 0.738 | 0.000 | 0.795 | <0.001 | 0.593 | 0.092 | 0.773 | <0.001 | |
| TG (mmol/L) | 0.269 | 0.124 | 0.180 | 0.317 | -0.315 | 0.447 | 0.216 | 0.311 | |
| TC (mmol/L) | 0.254 | 0.147 | 0.395 | 0.023 | 0.322 | 0.422 | 0.335 | 0.109 | |
| LDL-C (mmol/L) | 0.464 | 0.008 | 0.489 | 0.006 | 0.331 | 0.424 | 0.458 | 0.037 | |
| HDL-C (mmol/L) | -0.248 | 0.164 | 0.204 | 0.263 | 0.354 | 0.390 | 0.183 | 0.404 | |
| APOE (mg/dL) | 0.310 | 0.084 | 0.496 | <0.001 | 0.521 | 0.150 | 0.448 | 0.041 | |
| APOB (g/L) | 0.442 | 0.010 | 0.436 | 0.013 | 0.086 | 0.827 | 0.406 | 0.061 | |
| APOA1 (g/L) | -0.293 | 0.098 | 0.083 | 0.650 | -0.317 | 0.406 | 0.090 | 0.691 | |
| T (nmol/L) | 0.246 | 0.136 | 0.347 | 0.035 | -0.118 | 0.729 | 0.377 | 0.063 | |
| FSH (mIU/L) | 0.153 | 0.358 | 0.103 | 0.543 | 0.504 | 0.114 | 0.116 | 0.382 | |
| LH (mIU/L) | 0.081 | 0.631 | 0.036 | 0.834 | 0.348 | 0.295 | 0.036 | 0.866 | |
| LH/FSH | -0.049 | 0.769 | -0.052 | 0.760 | -0.091 | 0.790 | -0.065 | 0.757 | |
| E2 (pg/ml) | -0.148 | 0.376 | -0.145 | 0.393 | 0.669 | 0.024 | -0.337 | 0.099 | |
| P (ng/ml） | -0.226 | 0.172 | -0.229 | 0.172 | -0.271 | 0.420 | -0.333 | 0.103 | |
| PRL (ng/ml） | -0.477 | 0.002 | -0.432 | 0.008 | -0.293 | 0.382 | -0.540 | 0.005 | |
| SHBG (nmol/L) | -0.521 | 0.004 | -0.279 | 0.159 | -0.924 | 0.003 | -0.155 | 0.527 | |
| DHEA-S (µg/dl) | 0.081 | 0.700 | 0.202 | 0.343 | 0.836 | 0.078 | 0.465 | 0.052 | |
| 17α-OHP (ng/mL) | 0.155 | 0.554 | 0.098 | 0.718 | 0.855 | 0.145 | 0.103 | 0.763 | |
| AMH (ng/mL) | -0.260 | 0.314 | -0.248 | 0.354 | -0.938 | 0.062 | -0.123 | 0.718 | |
| WBC (10^9^/L) | 0.187 | 0.282 | 0.170 | 0.337 | 0.050 | 0.899 | 0.109 | 0.613 | |
| CRP (mg/L) | -0.016 | 0.929 | -0.041 | 0.824 | 0.209 | 0.619 | 0.062 | 0.779 | |
| \| TNF-α (pg/ml) \| \| --- \| | 0.241 | 0.279 | 0.121 | 0.603 | 0.962 | 0.176 | 0.029 | 0.912 | |
| IL-6 (pg/ml) | 0.455 | 0.033 | 0.251 | 0.273 | -0.474 | 0.686 | 0.195 | 0.453 | |
| IL-8 (pg/ml) | 0.308 | 0.187 | 0.138 | 0.573 | -0.447 | 0.705 | 0.063 | 0.824 | |

**Note:** Correlations between variables were analyzed by Spearman’s correlation test and an age-adjusted partial correlation test. Data were from participants without medication history of anti-diabetic, anti-hypertensive or anti-hyperlipidemia drugs.

**Abbreviation:** EHCT, euglycemic-hyperinsulinemic clamp test; SHBG, sex hormone-binding globulin; DHEA-S, dehydroepiandrosterone-sulfate; 17α-OHP, 17-α-hydroxyprogesterone; AMH, anti-Mullerian hormone; WBC, white blood cell count; CRP, c-reactive protein; TNF-α, tumor necrosis factor-α; IL-6, interleukin-6.

Supplementary Table 7: Association of plasma asprosin with T2DM or PCOS in fully adjusted models.

| Model adjustment | T2DM | | PCOS | |
| --- | --- | --- | --- | --- |
|  | OR, 95% CI | P | OR, 95% CI | P |
| Age | 2.219 (1.461-3.372) | <0.001 | 1.684 (1.102-2.575) | 0.016 |
| Age, BMI | 2.304 (1.487-3.570) | <0.001 | 1.776 (1.074-2.935) | 0.025 |
| Age, BMI, WBC | 2.283 (1.453-3.586) | <0.001 | 1.958 (1.125-3.411) | 0.018 |
| Age, BMI, WBC, SBP, DSP | 2.305 ((1.440-3.689) | <0.001 | 2.232 (1.189-4.193) | 0.013 |
| Age, BMI, WBC, SBP, DSP, HDL, TG, LDL, TC | 2,193 (1.319-3.648) | 0.002 | 2.859 (1.158-7.058) | 0.023 |
| Age, BMI, WBC, SBP, DSP, HDL, TG, LDL, TC, ALT, AST | 3.017 (1.366-6.662) | 0.006 | 2.542 (0.963-6.711) | 0.060 |
| Age, BMI, WBC, SBP, DSP, HDL, TG, LDL, TC, ALT, AST, γ-GGT | 3.306 (1.079-8.545) | 0.035 | 2.393 (0.883-6.485) | 0.086 |

**Note:** Results of binary logistic regression analyses are presented. Data were from participants without medication history of anti-diabetic, anti-hypertensive or anti-hyperlipidemia drugs.

**Abbreviation:** OR, odds ratio; CI, confidence interval.


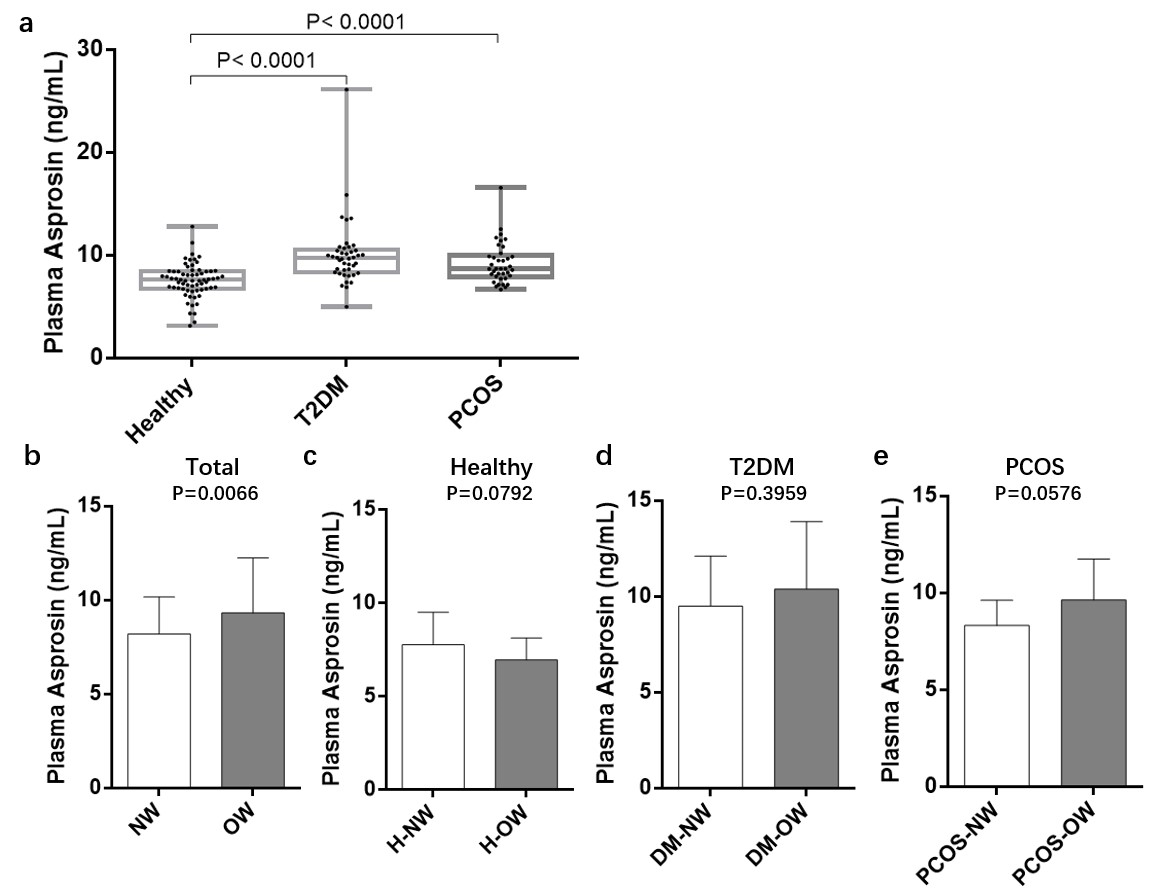
Supplementary Figure 1: Plasma asprosin concentrations in groups and the overweight/obese subgroups.

[Normal weight (NW) was defined as BMI < 24 kg/m^2^, and overweight/obesity (OW) was defined as BMI ≥ 24 kg/m^2^]. Data are presented as the means ± SD, and unpaired T tests were performed. Data are from participants without medication history of anti-diabetic, anti-hypertensive or anti-hyperlipidemia drugs.
